# A Recyclable, Metal-Free Mechanochemical Approach for the Oxidation of Alcohols to Carboxylic Acids

**DOI:** 10.3390/molecules25020364

**Published:** 2020-01-16

**Authors:** Kendra Leahy Denlinger, Preston Carr, Daniel C. Waddell, James Mack

**Affiliations:** Department of Chemistry, University of Cincinnati, 301 Clifton court, Cincinnati, OH 45221-0172, USA; denlingerk@xavier.edu (K.L.D.); prestoncarr25@gmail.com (P.C.); waddeldl@ucmail.uc.edu (D.C.W.)

**Keywords:** mechanochemistry, polymer supported, tempo, oxidation, recycle

## Abstract

The oxidation of primary alcohols under mechanochemical conditions in a Spex8000M Mixer/Mill was investigated. To facilitate ease of separation and recyclability, a polystyrene-bound version of a TEMPO catalyst was employed. When paired with Oxone^®^ in a stainless-steel vial with a stainless-steel ball, several primary alcohols were successfully oxidized to the corresponding carboxylic acids. The product was isolated using gravity filtration, which also allowed for the polystyrene-bound TEMPO catalyst to be recovered and reused in subsequent oxidation reactions. Furthermore, it was demonstrated that the size and steric hindrance of the primary alcohol does not hinder the rate of the reaction. Finally, the aldehyde was selectively obtained from a primary alcohol under ball milling conditions by using a combination of non-supported TEMPO with a copper vial and copper ball.

## 1. Introduction

The emerging field of mechanochemistry has garnered a lot of attention over the last few years [1,2,3,4,5,6,7,8]. This unique solvent-free approach has been applied to various areas of science with exciting results, specifically in the area of oxidation reactions [9,10,11,12,13,14,15,16,17,18,19]. Since mechanochemical reactions are solvent free by nature, these novel conditions would be complementary to reactions that are troublesome in solution. One field that seems to be tailor-made for mechanochemistry is the field of polymer-supported reagents (e.g., Merrifield resins). Merrifield resins have been very valuable to the field of peptide synthesis [20,21]. Conversely, the problems related to the swelling of these resins in solution have limited its potential in other areas of synthetic chemistry [22]. One area where polymer-supported resins have a vast amount of potential is the field of catalysis [23]. Catalysis is a very important area of chemistry that has increased in scope over the last 50 years. However, many catalysts are expensive, sensitive to air and/or moisture, and/or not commercially available. In addition, upon completion of the reaction, the catalyst is often difficult to recover and reuse. On the other hand, many polymer-supported catalysts are commercially available and, in principle, would allow for the complete recovery and reuse of the catalyst at the conclusion of the reaction. Although catalysts can be purchased or synthesized on a solid support, the swelling efficacy is a hindrance, thus preventing these reagents from reaching their full potential. We and others have previously shown that under mechanochemical conditions there are new mechanistic pathways to access the active sites of Merrifield resins [24,25,26,27,28]. Furthermore, since mechanochemical reactions are typically performed in the absence of a solvent, this methodology lends itself readily to the development of environmentally benign reactions. The combination of mechanochemistry and polymer-supported catalysts will allow for the development of catalytic conditions that are new, solvent free and completely recyclable.

The oxidation of alcohols typically requires the use of an oxidant and a catalyst to be successful, however many of these conditions are not considered environmentally benign [29,30,31]. Therefore, developing conditions in an environmentally benign manner has been important in the area of green chemistry [32,33,34,35]. The oxidation of alcohols is also important in medicinal chemistry. Pfizer, for example, has published a solvent selection guide as well as an introductory reagent selection guide [36]. One of the four reactions examined in this article was the oxidation of primary alcohols to aldehydes. Several different reaction conditions were examined and placed into the appropriate position on a Venn diagram, according to their ability to meet criteria in the areas of scalability, wide utility, and greenness. Reaction conditions utilizing TEMPO (i.e., 2,2,6,6-tetramethyl-piperidine-1-oxyl)/NaOCl fell into the middle of the Venn diagram, meeting the criteria of all three categories. Though there are many options for the oxidation of alcohols, most do not meet the criteria for Pfizer’s greenness category, indicating that there is still much work to be done in this area of green organic chemistry. Even though there are several alternatives to using toxic metals in the oxidation of alcohols to aldehydes, there are few metal-free methods in the literature for the conversion of an alcohol to a carboxylic acid. Of the metal-free variants in the literature, many use catalysts that are not commercially available and typically require column chromatography in the isolation step. 

Bolm and co-workers reported on the TEMPO oxidation of primary alcohols to aldehydes in the presence of Oxone^®^ (i.e., Potassium peroxymonosulfate) [37]. This report demonstrated a clean and facile conversion of alcohols to aldehydes in the presence of a quaternary ammonium salt using a biphasic medium. It has been shown that under solvent-free mechanochemical conditions, aldehydes can be readily converted to the corresponding carboxylic acids in the presence of Oxone^®^ [38]. The oxidation of primary alcohols to the corresponding carboxylic acids has also been accomplished using polymer-supported TEMPO in solution; however, under these conditions an organic solvent was needed in the reaction and/or at the purification stage [39,40,41]. We envisioned that the combination of a polystyrene bound sulfonic ester linked 2,2,6,6-tetramethyl-piperidine-1-oxyl species (hereafter referred to as PS-TEMPO) in the presence of Oxone^®^ under mechanochemical conditions would provide environmentally benign conditions that reduce the use of undesired solvents, chromatography-free, metal-free, and recyclable for the oxidation of alcohols to carboxylic acids. 

## 2. Results and Discussion

**Scheme 1:**
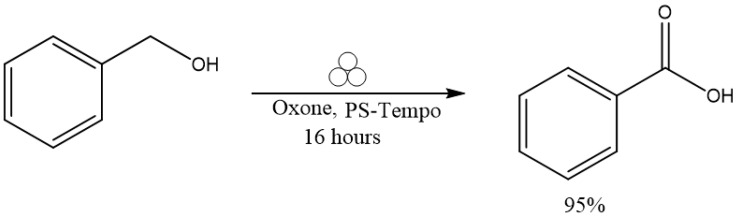
Oxidation of benzyl alcohol to benzoic acid with polymer supported TEMPO.

We started our investigation by milling benzyl alcohol with 1 equivalent of PS-TEMPO in the presence of 1 equivalent of Oxone^®^ for 16 h in a stainless-steel vial with a 3/16” stainless steel ball. We chose to use a stoichiometric amount of PS-TEMPO because it allows us to increase the reaction rate without increasing the cost by using the recovered catalyst in subsequent trials. These initial conditions would give us a broad baseline by which to compare other substrates that may be sensitive to less than stoichiometric equivalents and shorter reaction times. At the conclusion of the reaction, we washed the crude reaction mixture with acetone and filtered the pulverized polymer support, which then could be recycled and used in subsequent reactions. After removing the solvent, we observed a near quantitative yield to the pure benzoic acid (Scheme 1). 

In order to determine if the metal from the stainless steel had any effect on the reaction conditions, we conducted the same reaction in a Teflon vial with a 1/4” Teflon ball. Demonstrating that the metal from the stainless steel is not needed for oxidation to occur, we observed a 52% yield of the alcohol to the carboxylic acid by conducting the reaction in Teflon for 16 h. From previous work in the Mack group [42], this lower percent conversion was anticipated because Teflon is not as hard as stainless steel. When benzyl alcohol is milled with PS-TEMPO in the absence of Oxone^®^, we did not observe any formation of benzoic acid. Likewise, when benzyl alcohol is milled in the presence of Oxone^®^ without PS-TEMPO, we also did not observe any benzoic acid. This shows that both the PS-TEMPO and Oxone^®^ are needed for the oxidation to occur, which was expected based on the proposed mechanism of the reaction [43]. To study the scope of our reaction conditions, we extended our method to various other alcohols (Table 1), including both solid and liquid alcohols. 

**Figure 1:**
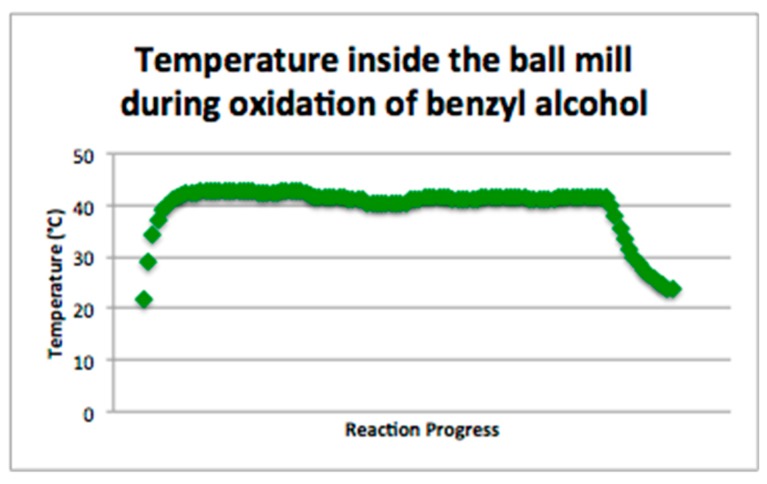
Temperature inside the ball mill during oxidation of benzyl alcohol.

The success of the solid alcohol oxidations indicates the true solid-phase nature of the system. The melting points for the solid alcohols are: 4-methylbenzyl alcohol m.p.: 59–61 °C; 4-bromobenzyl alcohol m.p.: 75–77 °C; 4-chlorobenzyl alcohol m.p.: 68–71 °C; and 4-nitrobenzyl alcohol m.p.: 92–94 °C. The temperature of the system was monitored as the reaction progressed using an iButton, which is shown in Figure 1. The maximum temperature reached during the reaction was 43 °C, and the system stayed between 41 °C and 43 °C for the duration of the 16-h reaction (Figure 1). Because the melting temperatures of the substrates are well above the temperature of the system (Figure 1), we can conclude that the reaction mixture in the vial is not a melt but rather solids interacting with other solids. Regarding the reactivity, when strongly electron donating groups are placed on the aryl ring system, the major product is the corresponding phenol, not the desired carboxylic acid. This is consistent with the results of a Dakin oxidation that have been observed in the literature with similar systems [39,44,45,46,47]. 

The temperature of the system was monitored as the reaction progressed using an iButton. A graph is shown in Figure 1. The maximum temperature reached during the reaction was 43 °C, and the system stayed between 41 °C and 43 °C for the duration of the 16-h reaction (Figure 1).

We have previously shown that the combination of mechanochemistry and polymer supports can significantly increase the greenness of a reaction. Using the Ecoscale [48], we compared a solution Wittig reaction with unsupported triphenylphosphine to a ball-milled Wittig reaction with polymer-supported triphenylphosphine; the reaction conditions went from inadequate on the EcoScale to excellent [49]. In this case, using mechanochemistry and the polymer-supported TEMPO as a substitute for the non-supported TEMPO in solution, the EcoScale improved from adequate to excellent.

**Figure 2:**
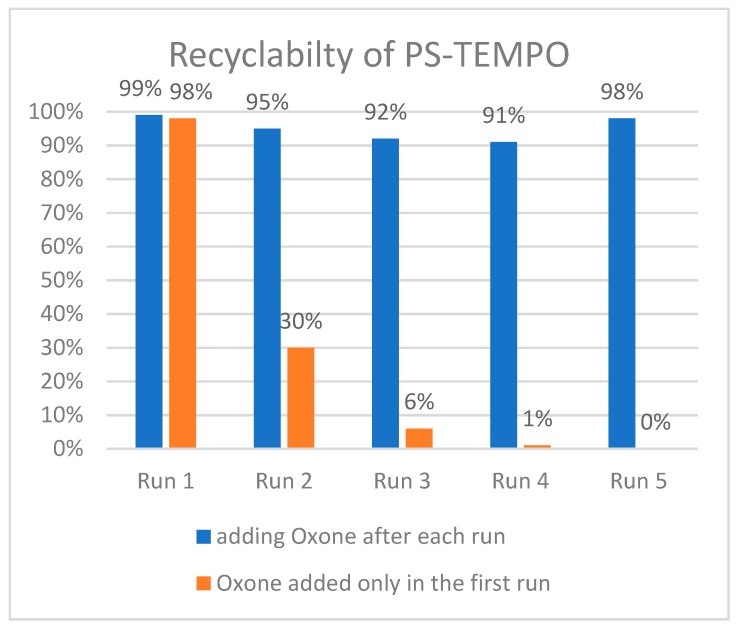
Recyclability study of PS-TEMPO.

After testing the initial reaction conditions, we wanted to determine the recyclability of the polymer-supported TEMPO catalyst. Starting with benzyl alcohol, we conducted four subsequent oxidation reactions, each time using the polymer-supported TEMPO from the previous run and charging the reaction with fresh Oxone^®^. We observed greater than 90% conversion of the benzyl alcohol to benzoic acid in each run (Figure 2). 

We also wanted to test whether we needed to charge the reaction with Oxone^®^ after each run. After conducting the recyclability study without the addition of Oxone^®^, we observed limited conversion of the alcohol to the carboxylic acid. It is clear that although the polymer-supported catalyst can be reused, a fresh load of Oxone^®^ is needed for every run. 

When Merrifield resins are swollen by a solvent, it allows the substrate greater accessibility to the active sites inside the polymer support. However, this mode of reactivity can be dependent on the size of the substrate, often requiring longer reaction times for larger substrates [50,51,52,53,54]. Under mechanochemical conditions, the polymer resin is pulverized; thus, the active sites of the polymer should be completely exposed to the substrate. Therefore, the reaction rate should be completely independent of the size of the substrate. To test this attribute, we compared the reaction rates of 1-octanol, 1-dodecanol, and 1-adamantanemethanol in order to study the effect the size of substrate has on the rate of reactivity. One equivalent of a base, K_2_CO_3_, was added to the linear aliphatic alcohol oxidations in order to suppress ester side product formation. Oxone^®^ itself creates an acidic environment, so without the addition of the base, the carboxylic acid product reacted with the alcohol starting material to form an ester. It is important to note that when aryl alcohols were oxidized additional base was not needed. We believe this is because the rate of aryl alcohols is faster than aliphatic alcohols, limiting the opportunity of the free alcohol to participate in an esterification. After adding the base, the ester side product was successfully suppressed, and we observed very little difference between the rates of reaction of the various-sized substrates compared to the reaction with non-supported TEMPO (Table 2) [55]. This suggests the size of the substrate has very little effect on the rate of reaction.

**Scheme 2:**
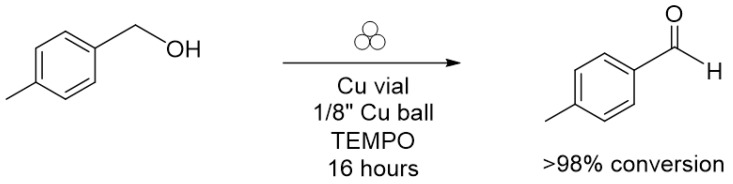
Conversion of primary alcohol to aldehyde using TEMPO in a copper vial.

We are also investigating selectively oxidizing the 4-methylbenzyl alcohol to the corresponding aldehyde. Based work by Stahl and co-workers, we hypothesized that copper, in the presence of oxygen, could catalyze the oxidation instead of Oxone^®^ [56]. We tested this theory using a copper vial as the copper source. No conversion was obtained with PS-TEMPO, however, quantitative conversion to the corresponding aldehyde was obtained when non-supported TEMPO was used under these conditions (Scheme 2). 

We hypothesize that there may be structural differences regarding non-supported TEMPO versus PS-TEMPO which do not allow the PS-TEMPO to interact with the copper vial effectively. This investigation is ongoing. 

## 3. Materials and Methods 

Deuterated chloroform was obtained from Cambridge Isotope Laboratories Inc. (Andover, MA. USA), and used without further purification. TEMPO functionalized polystyrene, 2% cross-linked with divinylbenzene, (PS-TEMPO) was obtained from Biotage**^®^** (Uppsala, Sweden) and used without further purification. 1-Dodecanol was obtained from Aldrich (St. Louis, Missouri, MO, USA) and used without further purification. Phenethyl alcohol was obtained from Matheson Coleman & Bell (MCB) (Washington, DC, USA) and used without further purification. Other alcohols and oxidants were obtained from Fisher Scientific (Hampton, New Hampshire, NH, USA) and used without further purification. ^1^H NMR spectroscopy was performed using a Bruker Avance 400 spectrometer (Billerica, Massachusetts, MA, USA). The temperature of the vial was determined by use of a temperature logging “iButton” (DS1922E-F5#, Maxim Integrated Circuits, San Jose, California, CA, USA http://www.ibutton.com) clamped against the vial. This temperature rises for approximately the first hour and then maintains for the remainder of the experiment. 

### 3.1. PS-TEMPO/Oxone^®^/Stainless Steel 

To a customized 3.0 mL stainless steel vial was added 250 mg (0.99 mmol/g) of PS-TEMPO, 0.314 g Oxone**^®^** (0.5 mmol), and 0.25 mmol of alcohol. A 3/16” stainless steel ball was added to the vial. The vial was shaken at 18Hz for 16 h in a Spex8000M Mixer/Mill (Metuchen, NJ, USA). The resulting mixture was gravity filtered with one of three polar solvents (acetone, ethyl acetate, and ethanol), depending on the solubility of the desired product. The solvent was removed under reduced pressure, affording the carboxylic acid product. The PS-TEMPO was recovered, dried and used in the subsequent reactions. ^1^H NMR spectroscopy using a Bruker Avance 400 spectrometer was performed to assess the extent and purity of the reaction products.

### 3.2. Substrate Size Study

To a customized 3.0 mL stainless steel vial was added 250 mg (0.99 mmol/g) of PS-TEMPO, 0.314 g Oxone**^®^** (0.5 mmol), and 0.25 mmol of alcohol. For 1-octanol and 1-dodecanol, 0.25 mmol K_2_CO_3_ was also added to the vial. A 3/16” stainless steel ball was added to the vial. The vial was shaken at 18 Hz for 16 h in a Spex8000M Mixer/Mill. The resulting mixture was gravity filtered with one of three polar solvents (acetone, ethyl acetate, and ethanol), depending on the solubility of the desired product. The solvent was removed under reduced pressure, affording the product. The PS-TEMPO was recovered, dried and used in the subsequent reactions. ^1^H NMR spectroscopy using a Bruker Avance 400 spectrometer was performed to assess the extent and purity of the reaction products.

### 3.3. TEMPO/Copper

To a customized copper vial was added 0.25 mmol TEMPO and 0.25 mmol of alcohol. A 1/8” copper ball was added to the vial. The vial was shaken at 18 Hz for 16 h in a Spex8000M Mixer/Mill. The resulting mixture was gravity filtered with one of three polar solvents (acetone, ethyl acetate, and ethanol), depending on the solubility of the desired product. The solvent was removed under reduced pressure, affording a mixture of the aldehyde product and TEMPO. ^1^H NMR spectroscopy using a Bruker Avance 400 spectrometer was performed to assess the extent and purity of the reaction products. Please find ^1^H NMR Spectra of selected products in the Supplementary Materials.

## 4. Conclusions

In conclusion, we further demonstrated the powerful combination of mechanochemistry with polymer supported resins. These reaction conditions are a novel way to activate polymer supported reagents that are faster and more general than the traditional swelling mechanism. Additionally, due to the pulverization of the polymer, the rate of the reaction is not dictated by the size of the substrate but rather mostly by the milling process, which is much improved over the traditional solution based conditions. Furthermore, the nature of the reactions allowed us to develop a metal-free, recyclable, environmentally benign method for the conversion of alcohols to their corresponding carboxylic acids. We are currently applying these oxidation conditions to secondary alcohols for the oxidation of ketones and to determine the potential selectivity of primary over secondary alcohols. Mechanochemistry is a rapidly growing field and as we learn more about these unique reaction conditions, the more prominent they will become in the chemical literature.

## Figures and Tables

**Table 1:**
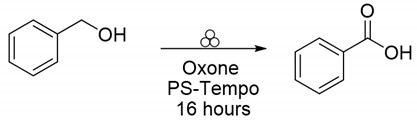
Oxidation of alcohols to carboxylic acids with polymer-supported TEMPO.

Entry	Alcohol	% Yield
1	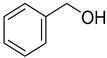	>98
2	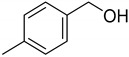	>98
3	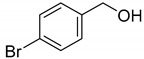	>98
4	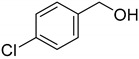	>98
5	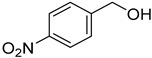	>98
6	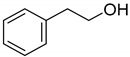	64
7	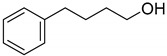	75
8	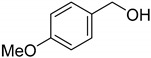	<5

**Table 2:**
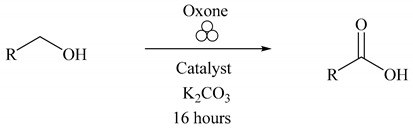
Comparison of PS-TEMPO with non-supported TEMPO.

Entry	Alcohol	Catalyst	% Conversion	Catalyst	% Conversion
1	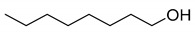	TEMPO	81	PS-TEMPO	95
2	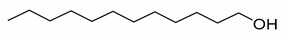	TEMPO	76	PS-TEMPO	95
3	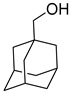	TEMPO	80 *	PS-TEMPO	89 *

* Recorded percent conversion includes both 1-adamantanecarboxylic acid and 1-adamantanol (produced analogously to the Dakin reaction as described above in the aryl system with a strong electron donating group) [16,20,21,22,23].

## Supplementary Materials

The following are available online at https://www.mdpi.com/1420-3049/25/2/364/s1, Figure S1: 1H NMR of substrates and calculation of EcoScale.
