# Supplementary material for: A Recyclable, Metal-Free Mechanochemical Approach for the Oxidation of Alcohols to Carboxylic Acids
Source: Molecules. 2020 Jan 16;25(2):364. doi: 10.3390/molecules25020364 (PMC7024246; doi:10.3390/molecules25020364)
Supplement: Supplementary file 1 [file molecules-25-00364-s001.pdf]

Electronic Supplementary Information

A recyclable, metal-free mechanochemical approach for the oxidation of alcohols to carboxylic acids

Kendra Leahy Denlinger, Preston Carr, Daniel C. Waddell and James Mack*

*Corresponding author
University of Cincinnati
301 Clifton Court
Cincinnati, OH USA

E-mail address: james.mack@uc.edu
Tel.: 1.513.556.9249
Fax: 1.513.556.9239

Contents:

^{1}H NMR Spectra of selected products…………………………………………………………………2

EcoScale Data……………………………………………………………………………………………11

^{1}H NMR Spectra of isolated Carboxylic Acid Products

All ^{1}H NMR spectra were obtained in $CDCl_3$ unless otherwise noted. Due to insolubility in $CDCl_3$ ^{13}C NMR was not taken of 4-bromobenzoic acid, 4-chlorobenzoic acid, 4-nitrobenzoic acid. Conversions in all reactions were measured by GC-MS. The conversion was calculated using the peak integrations of the retention times of the products against the starting alcohol (i.e., conversion= products (desired)/reactants and products (total). Errors in the conversion measurements were estimated by comparing the results of at least 3 integrations of each spectrum.

Benzoic Acid

^{1}H NMR ($CDCl_3$, 400 MHz, ppm): δ 7.48 (t, J = 8.0 Hz, 2H), 7.62 (t, J = 8.0 Hz, 1H), 8.12 (d, J = 8.0 Hz, 2H); ^{13}C NMR ($CDCl_3$, 100 MHz, ppm): δ 128.5, 129.2, 130.2, 133.9, 172.6.
NMR spectral data matched with previous report.[1]

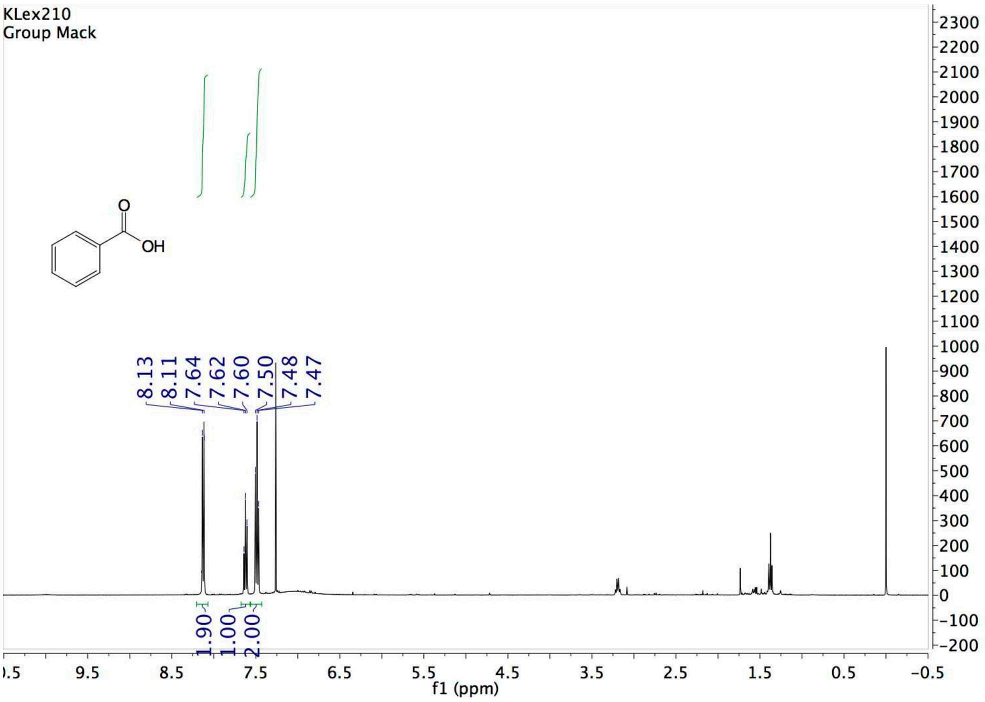

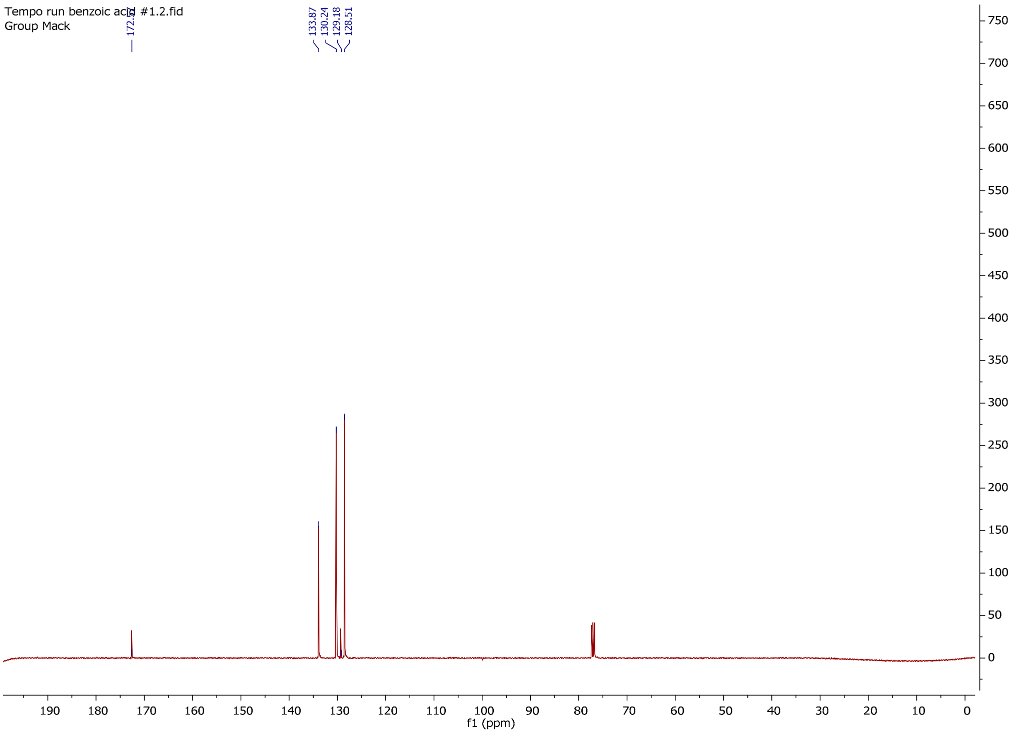

4-methylbenzoic acid

^{1}H NMR ($CDCl_3$, 400 MHz, ppm): δ 2.46 (s, 3H), 7.31 (d, J = 8.0 Hz, 2H), 8.02 (d, J = 8.0 Hz, 2H); ^{13}C NMR ($CDCl_3$, 100 MHz, ppm): δ 21.8, 126.6, 129.2, 130.3, 144.7, 172.5;
NMR spectral data matched with previous report.[2]

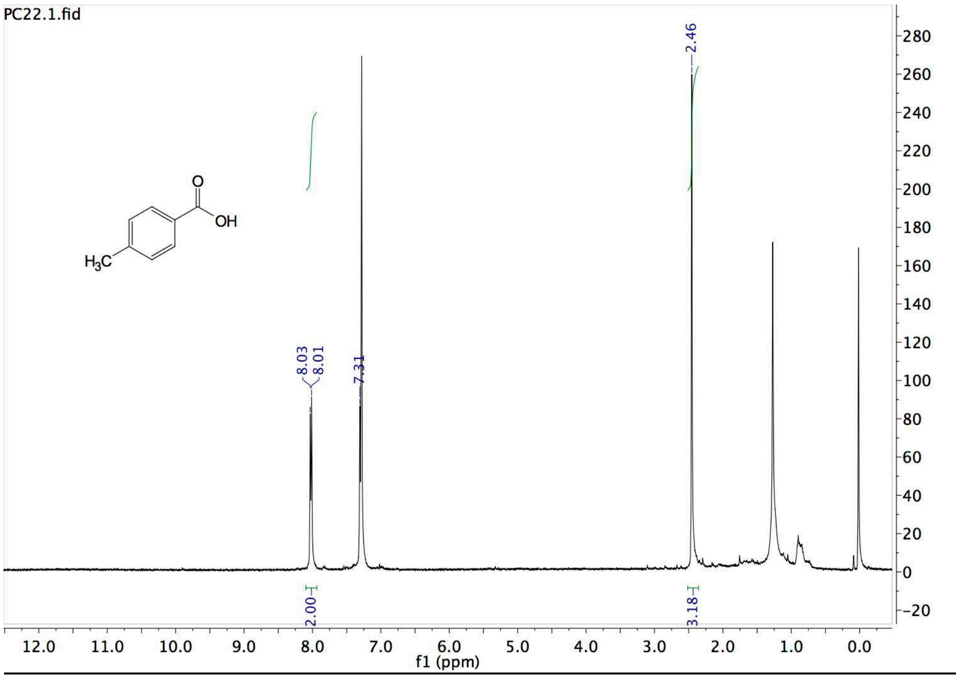

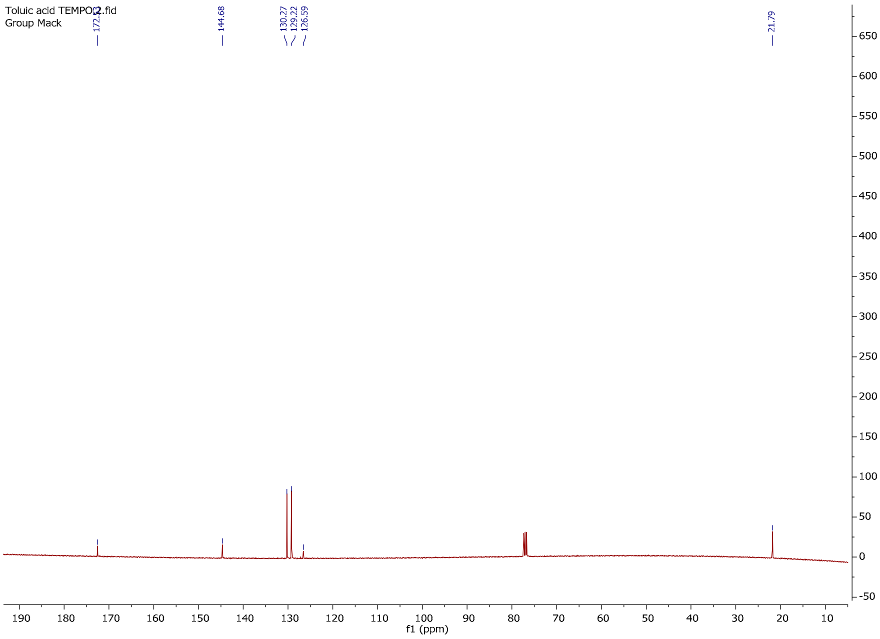

<u>4-Bromobenzoic acid</u>

^{1}H NMR (acetone-d_6, 400 MHz, ppm): δ 7.72 (d, J = 8.0 Hz, 2H), 7.97 (d, J = 8.0 Hz, 2H H); MS (m/e) 202 (M+•),
^{1}HNMR spectral data matched with previous report.[3]
MS data matched with previous report[4]

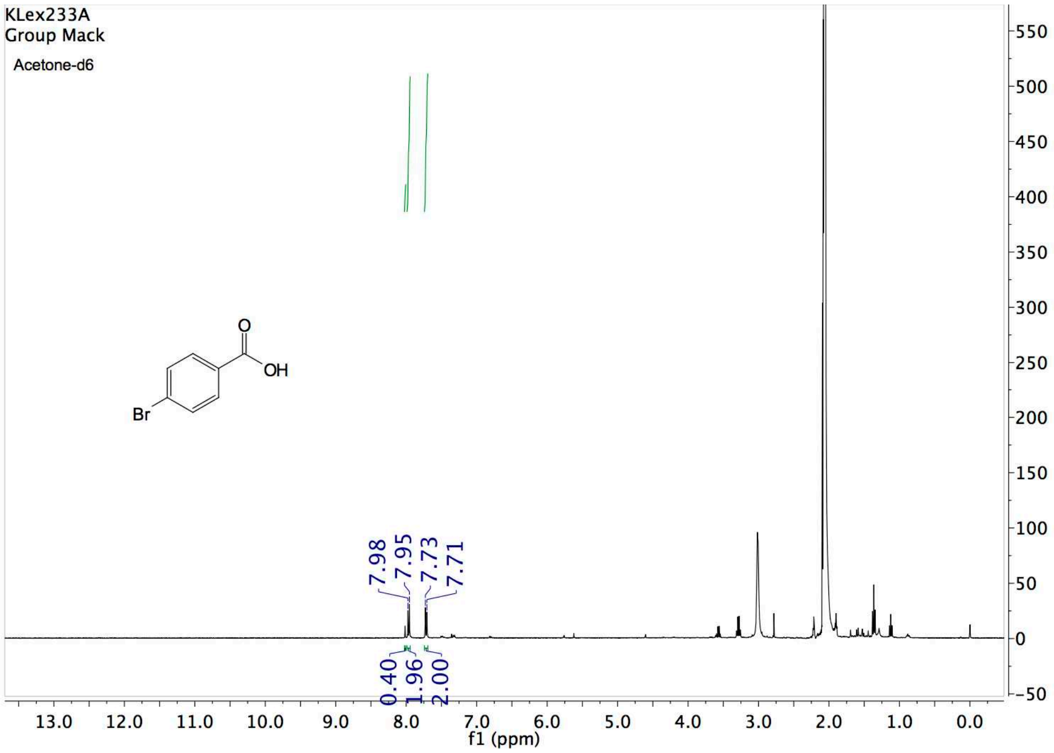

4-Chlorobenzoic acid

^{1}H NMR (acetone-d_6, 400 MHz, ppm): δ 7.56 (d, J = 8 Hz, 2H,), 8.04 (d, J = 8 Hz, 2H);
^{1}H NMR spectral data matched with previous report.[3]
MS data match with previous report.[4] MS (m/e) 156 (M+•),

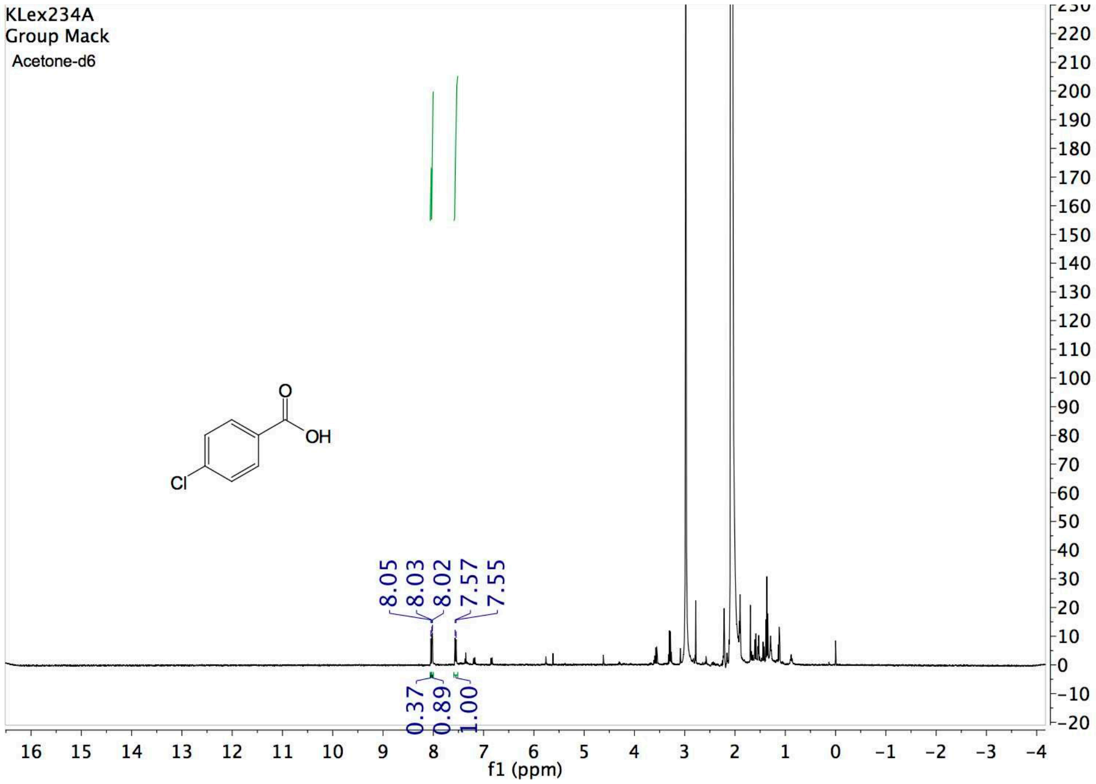

4-Nitrobenzoic acid

^{1}H NMR ($CDCl_3$, 400 MHz, ppm): δ 8.28 (d, J = 8 Hz, 2H), 8.33 (d, J = 8 Hz, 2H); MS (m/e) 166 [M-1] + .
^{1}H NMR spectral data matched with previous report.[2]
MS data matched with previous report[5]

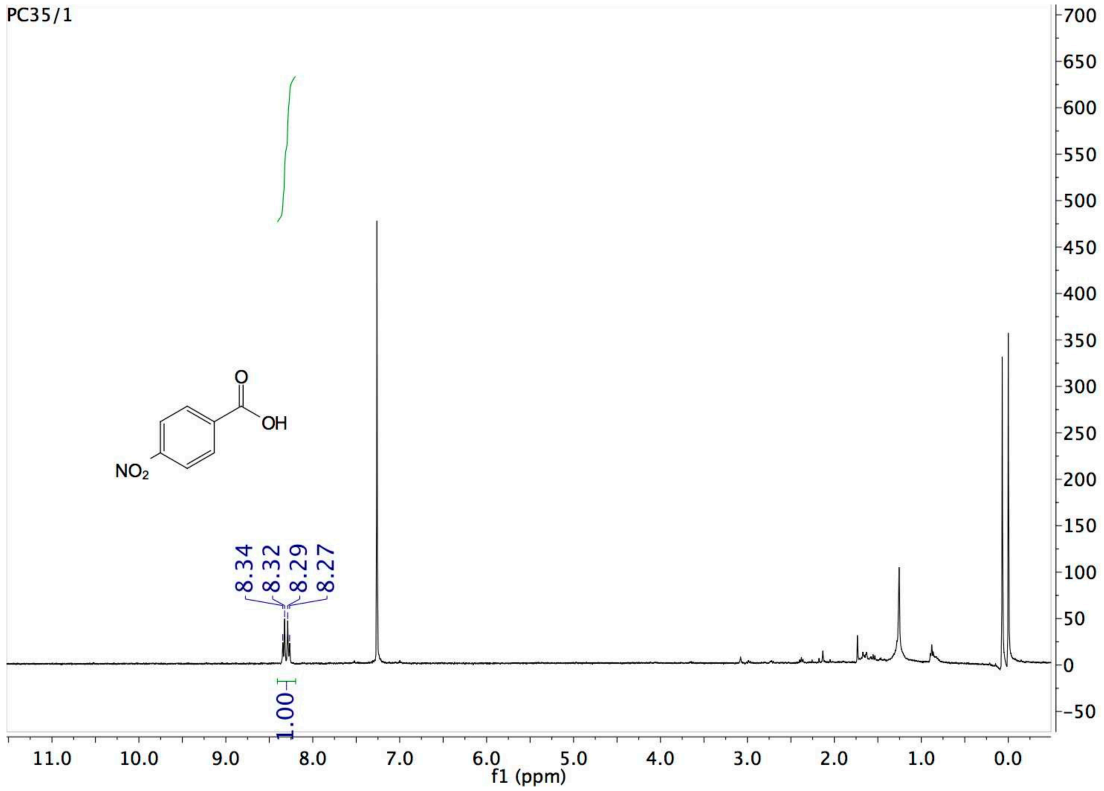

Phenyl acetic acid

^{1}H NMR ($CDCl_3$, 400 MHz, ppm): δ 3.64 (s, 3H), 7.29-7.48 (m, 5H), ^{13}C NMR ($CDCl_3$, 100 MHz, ppm): δ 41.1, 127.4, 128.7, 129.4, 133.2, 178.2; Spectrum data match with previous report.[6]

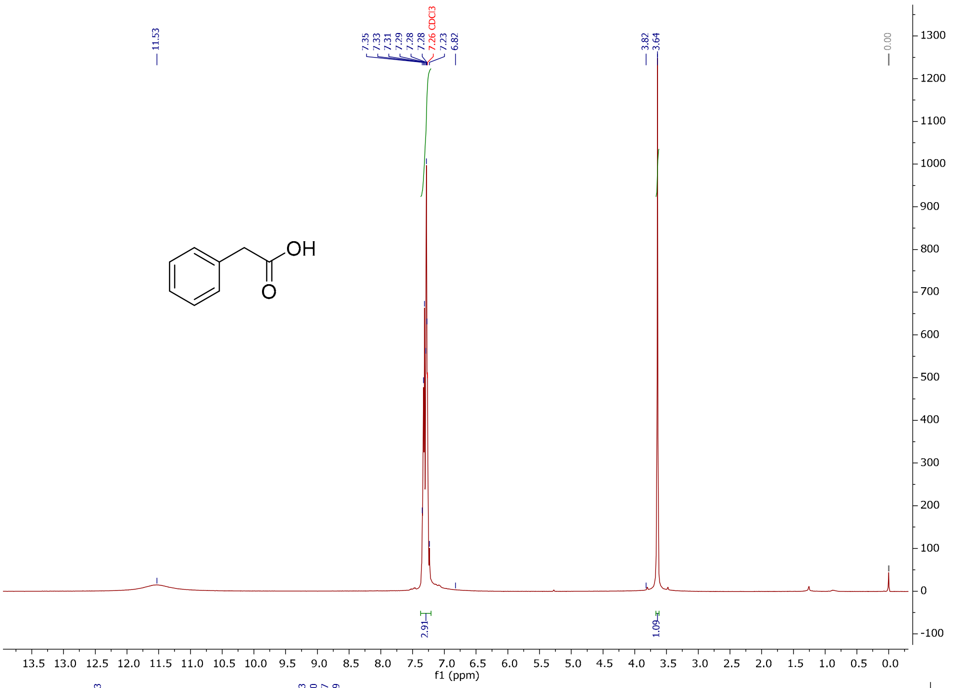

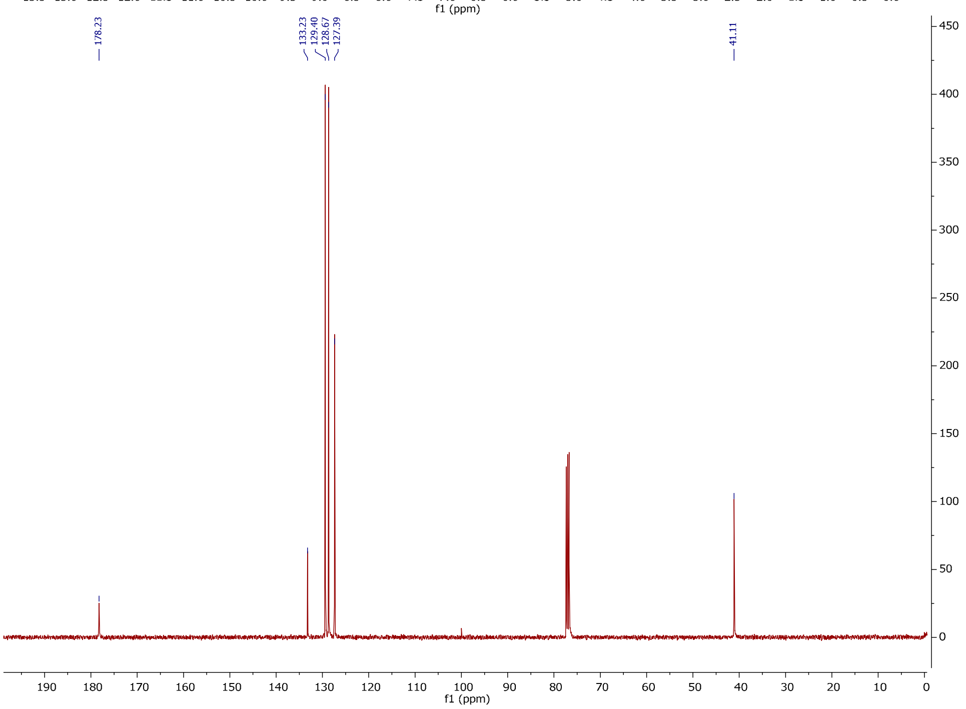

4-phenylbutanoic acid

^{1}H NMR ($CDCl_3$, 400 MHz, ppm): 1.97 (p, J = 8 Hz, 2H) , 2.38 (t, J = 8 Hz, 2H), 2.68 (t, J = 8 Hz, 2H) 7.15-7.25 (m, 3H), 7.26-7.35(m, 2H); ^{13}C NMR ($CDCl_3$, 100 MHz, ppm): δ 26.2, 33.3, 35.0, 126.1, 128.4, 141.2, 180.0;
NMR spectral data matched with previous report[7]

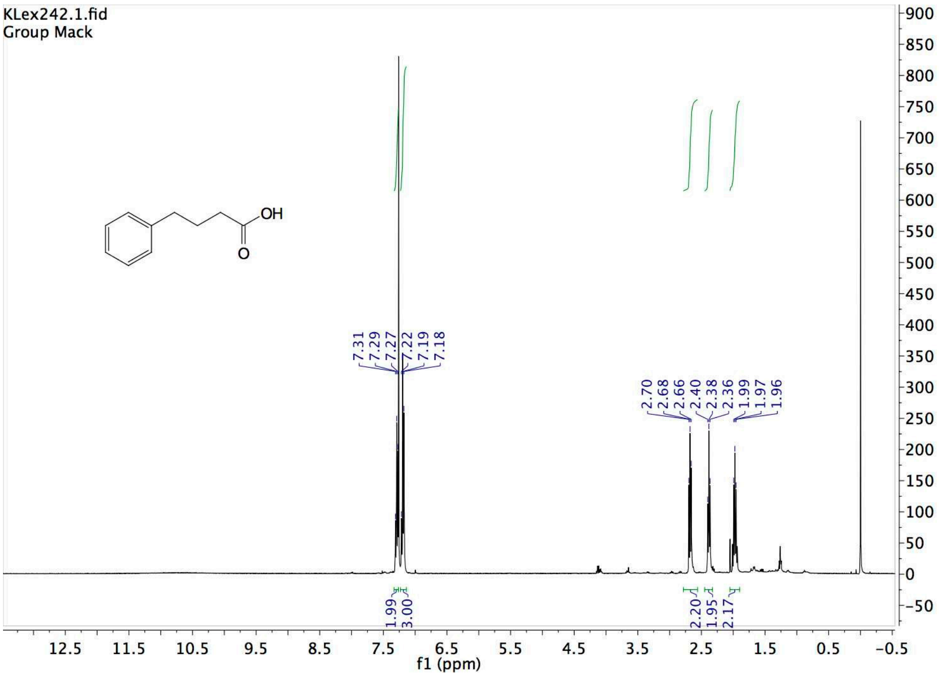

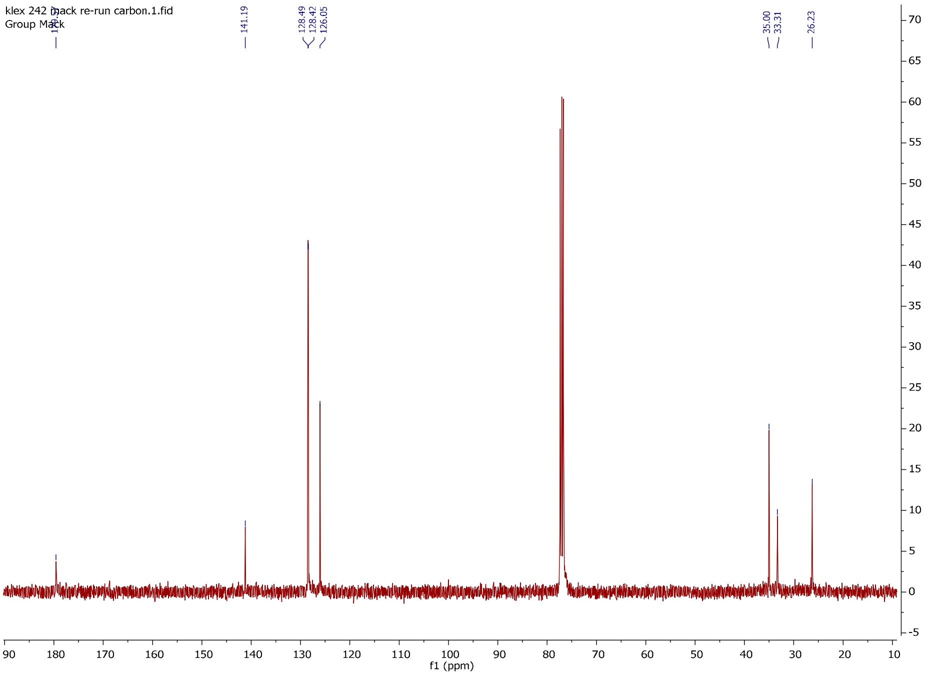

4-methylbenzaldehyde

^{1}H NMR ($CDCl_3$, 400 MHz, ppm): 2.38 (s, 3H) , 7.22 (d, J = 8 Hz, 2H), 7.73 (d, J = 8 Hz, 2H), 9.92 (s, 1H); ^{13}C NMR ($CDCl_3$, 100 MHz, ppm): δ 21.7, 129.6, 129.7, 134.1, 145.4. 191.9; NMR spectral data matched with previous report[8]

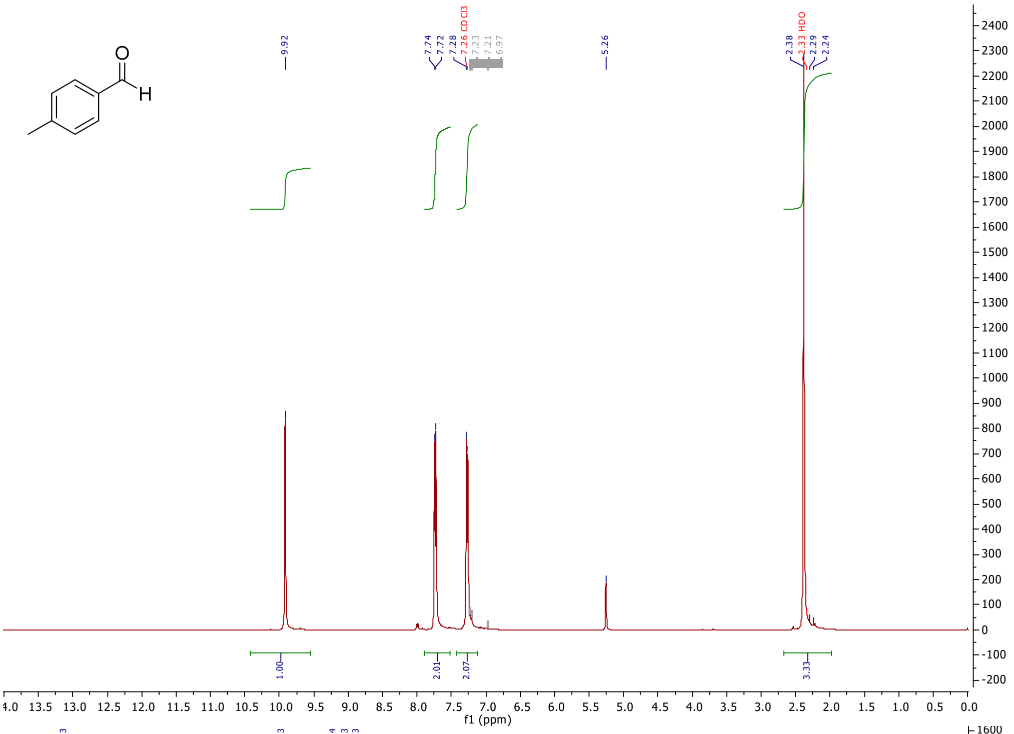

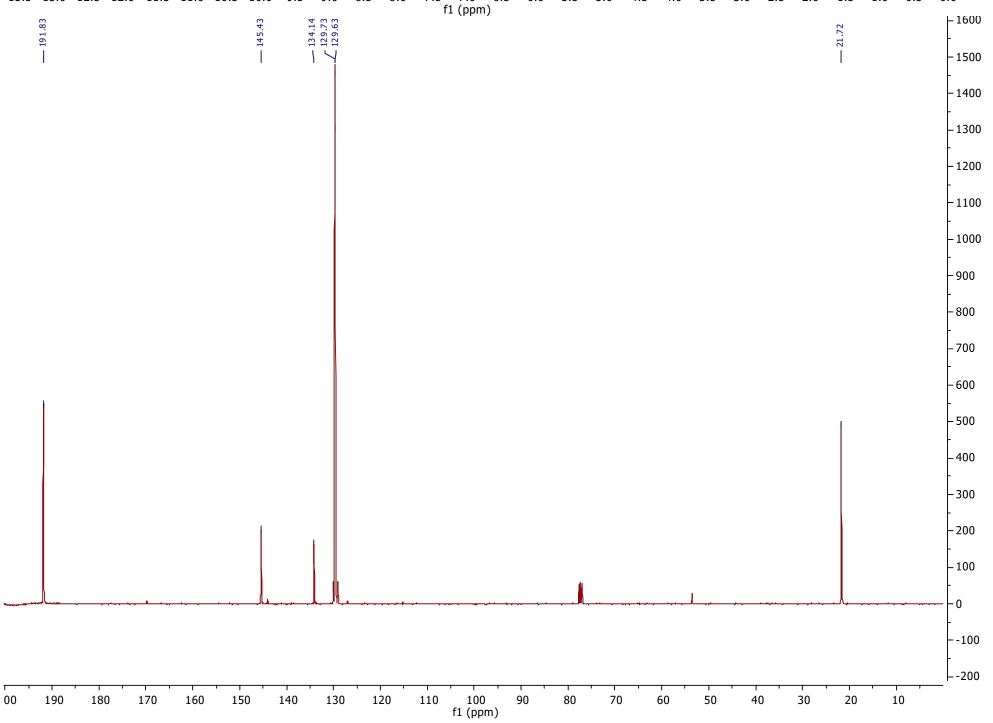

EcoScale calculations performed at: http://ecoscale.cheminfo.org/calculator

EcoScale for solution based reaction based on the following paper:
Carsten Bolm,* Angelika S. Magnus, and Jens P. Hildebrand, *Org. Lett.*, Vol. 2, No. 8, **2000**, 1173-1175.

Ecoscale calculator

Reagents

Link

	identifier*	name	MF*	MW	density	purity*	ml	g	mmoles	equiv.
1		Benzyl alcohol	C7H8O	108.13992	1.044	100%	0.103582	0.10814	1	0.0128200
2		2,2,6,6-Tetramethylpiperidinooxy	C9H18NO	156.24802		100%	0	0.001562	0.01	0.0001282
3		Dipotassium peroxymonosulfate	O6K2S	206.253		100%	0	0.453757	2.2	0.0282041
4		Tetrabutylammonium bromide	C16H36BrN	322.37254		100%	0	0.012895	0.04	0.0005128
5		Dichloromethane	CH2Cl2	84.93288	1.325	100%	5	6.625	78.002771	1
6		n-Hexane	C6H14	86.17716	0.659	100%	90	59.31	688.23340	8.8231916
7		Ethyl acetate	C4H8O2	88.10632	0.902	100%	10	9.02	102.37631	1.3124701

Products

identifier*:	name:	MF*:	MW:	g:	mmoles:	g theor:	yield:
	Benzaldehyde	C7H6O	106.12404	0		8.277969	0

Conditions

Reagents

Name	mmoles	eq.	Bp	Hazard	Price
Benzyl alcohol	Infinity	0.01	205		
2,2,6,6-Tetramethylpiperidinooxy	Infinity	0			
Dipotassium peroxymonosulfate	Infinity	0.02			
Tetrabutylammonium bromide	Infinity	0			
Dichloromethane	Infinity	1	39		
n-Hexane	Infinity	8.82	69		
Ethyl acetate	Infinity	1.31	75		

Parameter	Possible items	Selected items	Penalty
Yield	90		-5
Price / availability			-5
Safety			-15
Technical setup	Common set-up; Instruments for controlled addition of chemicals; Unconventional activation technique	Common set-up	0
Temperature / time	Heating, > 1h; Cooling to 0°C; Cooling, < 0°C	Room temperature, < 24h	-1
Workup and purification	Simple filtration; Removal of solvent with bp < 150°C; Crystallization and filtration; Removal of solvent with bp > 150°C	Classical chromatography; Removal of solvent with bp < 150°C	-10
**EcoScale**			64

EcoScale for our current methodology:

Ecoscale calculator

Reagents

Link

	identifier*	name	MF*	MW	density	purity*	ml	g	mmoles	equiv.
1		Benzyl alcohol	C7H8O	108.13992	1.044	100%	0.025896	0.027035	0.25	1
2		Dipotassium peroxymonosulfate	O6K2S	206.253		100%	0	0.103127	0.5	2
3		2,2,6,6-Tetramethylpiperidinooxy	C9H18NO	156.24802		100%	0	0.039062	0.25	1
4		Acetone	C3H6O	58.08004	0.79	100%	25	19.75	340.04797	1360.1919

Products

identifier*:	name:	MF*:	MW:	g:	mmoles:	g theor:	yield:
	Benzoic acid	C7H6O2	122.12344	0		0.030531	0

Conditions

Reagents

Name	mmoles	eq.	Bp	Hazard	Price
Benzyl alcohol	Infinity	1	205		
Dipotassium peroxymonosulfate	Infinity	2			
2,2,6,6-Tetramethylpiperidinooxy	Infinity	1			
Acetone	Infinity	1360.19	56		

Yield	95		-2.5
Price / availability			-5
Safety			-5
Technical setup	Possible items: Instruments for controlled addition of chemicals; Unconventional activation technique; Pressure equipment, > 1 atm; Any additional special glassware	Selected items: Unconventional activation technique	-2
Temperature / time	Possible items: Heating, > 1h; Cooling to 0°C; Cooling, < 0°C	Selected items: Room temperature, < 24h	-1
Workup and purification	Possible items: Adding solvent; Simple filtration; Removal of solvent with bp < 150°C; Crystallization and filtration	Selected items: Removal of solvent with bp < 150°C; Simple filtration	0
**EcoScale**			84.5

References

1. Dolui, P.; Hazra, S.; Deb, M.; Elias, A.J. Picolinamide Assisted Oxidation of CH2 Groups Bound to Organic and Organometallic Compounds Using Ferrocene as a Catalyst. *Organometallics* **2019**, *38*, 2015-2021, doi:10.1021/acs.organomet.9b00085.
2. Sathyanarayana, P.; Ravi, O.; Muktapuram, P.R.; Bathula, S.R. Copper catalyzed oxygen assisted C(CNOH)–C(alkyl) bond cleavage: a facile conversion of aryl/aralkyl/vinyl ketones to aromatic acids. *Organic & Biomolecular Chemistry* **2015**, *13*, 9681-9685, doi:10.1039/C5OB01569C.
3. Gong, D.; Hu, B.; Chen, D. Bidentate Ru(ii)-NC complexes as catalysts for the dehydrogenative reaction from primary alcohols to carboxylic acids. *Dalton Transactions* **2019**, *48*, 8826-8834, doi:10.1039/C9DT01414D.
4. Bjørsvik, H.-R.; Liguori, L.; Minisci, F. New Selective Oxidation Reactions by Nitroarenes in Basic Medium Involving Electron-Transfer Processes. *Organic Process Research & Development* **2001**, *5*, 136-140, doi:10.1021/op000095p.
5. Patil, V.V.; Shankarling, G.S. Steric-Hindrance-Induced Regio- and Chemoselective Oxidation of Aromatic Amines. *J Org Chem* **2015**, *80*, 7876-7883, doi:10.1021/acs.joc.5b00582.
6. León, T.; Correa, A.; Martin, R. Ni-Catalyzed Direct Carboxylation of Benzyl Halides with CO2. *J. Am. Chem. Soc.* **2013**, *135*, 1221-1224, doi:10.1021/ja311045f.
7. Jung, H.-Y.; Chang, S.; Hong, S. Strategic Approach to the Metamorphosis of γ-Lactones to NH γ-Lactams via Reductive Cleavage and C–H Amidation. *Organic Letters* **2019**, *21*, 7099-7103, doi:10.1021/acs.orglett.9b02673.
8. Zheng, J.; Lin, S.; Zhu, X.; Jiang, B.; Yang, Z.; Pan, Z. Reductant-directed formation of PS–PAMAM-supported gold nanoparticles for use as highly active and recyclable catalysts for the aerobic oxidation of alcohols and the homocoupling of phenylboronic acids. *Chem. Commun.* **2012**, *48*, 6235-6237, doi:10.1039/C2CC31948A.
